# Recognition of Delirium Superimposed on Dementia: Is There an Ideal Tool?

**DOI:** 10.3390/geriatrics8010022

**Published:** 2023-02-02

**Authors:** Priyanka Shrestha, Donna M. Fick

**Affiliations:** 1 Community of Policy, Populations and Systems, The George Washington University School of Nursing, Washington, DC 20006, USA; 2 The Ross and Carol Nese College of Nursing, The Pennsylvania State University, University Park, PA 16802, USA

**Keywords:** delirium, delirium superimposed on dementia, identifying delirium in dementia

## Abstract

Delirium in persons with dementia (DSD) is a common occurrence. Over the past three decades, several tools have been developed and validated to diagnose delirium, yet there is still a shortage of tools recommended in persons with dementia and there is a lack of sufficient research on the accuracy of performance of such tools in this growing population. The purpose of this article is to (1) conduct a clinical review of the detection of DSD across settings of care by formal health care professionals and informal family members and care partners; (2) identify barriers and facilitators to detection and highlight delirium tools that have been tested in person with dementia; and (3) make recommendations for future research, practice, and policy. Given this review, an “ideal” tool for DSD would point to tools being brief, easy to integrate into the EMR, and accurate with at least 90% accuracy given the poor outcomes associated with delirium and DSD. Knowing the baseline and communication between family members and healthcare professionals should be a top priority for education, research, and health systems policy. More work is needed in better understanding DSD and optimizing and standardizing feature assessment, especially the acute change feature at the bedside for DSD.

## 1. Introduction

Delirium is a serious and potentially life-threatening condition that is characterized by a sudden onset of confusion, altered consciousness, and changes in behavior that has an underlying cause not explained by a pre-existing cognitive impairment [1]. Delirium is common in older adults, under-detected, and associated with poor short- and long-term outcomes. The frequency of delirium alone ranges from 11 to 51%, with much higher rates with increasing age, pre-existing cognitive impairment, and in certain settings such as surgical, intensive care unit (ICU), and long-term care [2,3]. Delirium or altered mental state is a cause of hospital admission for over 40% of persons living in a nursing home and is as high as 75% in the ICU settings [4,5]. Delirium in persons with pre-existing dementia, commonly known as delirium superimposed on dementia (DSD), is much more common than delirium alone. DSD occurs in over half of all hospitalized older adults with dementia, with even higher incidence rates with increasing dementia severity [6,7]. A study conducted on 104 older adults who were at least aged 65 years and admitted to a medical or geriatric unit found 22% of patients diagnosed with early-stage dementia developed delirium during their stay. Also, the incidence of delirium increased with the severity of dementia (i.e., 45% in mid-stage dementia and 58% in late-stage dementia) [8]. Across different specialities, the prevalence of DSD is the highest among orthopedic (63.2%) and general surgery inpatients (62.3%) [7].

Delirium is unrecognized in 60–70% of older adult patients, with hypoactive delirium and DSD most likely to be missed [2,9,10]. DSD that is not detected often results in serious complications including prolonged length of hospital stay, poorer cognitive and functional outcomes, increased risk of nursing home placement, and mortality compared with persons with dementia alone [6,7]. Furthermore, DSD is associated with adverse effects in family care partners, including anxiety, depression, burnout, and higher healthcare costs that can be 52% higher when dementia is considered [6,11,12]. Studies have found that a one-year onset delay of dementia would substantially decrease the prevalence of dementia [13]. In addition, the reduction of other comorbidities, especially delirium, can aid in alleviating the progression of dementia and delay the diagnosis of dementia in cognitively normal or mildly impaired older adults [14,15]. Lack of early detection of delirium in persons with dementia is a missed opportunity for delirium prevention and management, which is potentially an important preventative factor for the incidence and delay of the progression of dementia [16,17]. The delay in the unmasking of incident cases of dementia and timely management of delirium in older adults with dementia may significantly reduce the burden to persons living with dementia (PLWD), family care partners, clinicians, and health care systems [14]. Hence, to understand the current state of science in the detection of DSD, the purpose of this article is threefold: (1) to conduct a clinical review of the detection of DSD across settings of care by formal health care professionals and informal family members and care partners, (2) to identify barriers and facilitators to detection and highlight delirium tools that have been tested in person with dementia, and (3) to make recommendations for future research, practice, and policy. To identify the most relevant articles, our methods included searching PubMed, the Cumulative Index to Nursing and Allied Health Literature (CINAHL), ProQuest, and hand searching the Network for Investigation of Delirium Unifying Scientists (NIDUS) bibliography of review and validation papers of detection and recognition of DSD for the past 10 years. This was not an exhaustive or systematic review.

## 2. Barriers and Facilitators to Recognition

Delirium and dementia are amongst the most common cognitive disorders encountered by clinicians in both hospital and community settings, and the prevalence is rising as the ageing population is growing worldwide. Although highly preventable and reversible, the recognition of delirium, especially in persons with dementia, is challenging for clinicians, which is not a new finding [18,19,20]. In a study by Inouye and colleagues, more than half of the clinicians (physicians: 65%; nurses: 43%) missed delirium in hospitalized older patients [21]. The factors leading to poor recognition of DSD share similar issues with delirium alone, especially in regards to system issues, but DSD is also complicated by additional factors.

First, there is not an alignment with diagnostic criteria, cognitive tools, and guidelines. The diagnosis of delirium is dependent on the diagnostic criteria of the International Statistical Classification of Diseases and Related Health Problems, 10th Revision (ICD-10), and the Diagnostic and Statistical Manual of Mental Disorders, Fifth Edition (DSM-5) [1]. However, to date there are no specific criteria to assist health care professionals (HCPs) with recognition and diagnosis. Also, cognitive tests are generally used to identify delirium in older adults. Scores in such cognitive tests could be confounded in older adults with pre-existing dementia, regardless of whether they have delirium, further contributing to the challenge of DSD diagnosis [22]. Another factor that may contribute to the challenges of assessing delirium in persons with dementia are the different causes of dementia and types of dementia, such as Lewy body dementia or vascular dementia. Most studies do not measure or differentiate between types of dementia and the accuracy of tools for DSD are likely to vary by type or underlying causes of dementia [23]. Currently, there are no specified clinical practice guidelines for the timely recognition and management of delirium in the United States [24]. There are well recognized clinical practice guidelines, such as National Institute for Health and Care Excellence (NICE) and Scottish Intercollegiate Guidelines Network (SIGN) developed in the U.K., suggesting treating delirium first in cases of difficulty diagnosing between delirium, dementia, or DSD. However, this suggestion is often not utilized in clinical practice due to the lack of knowledge and complexity of DSD presentation [24].

Second, providers are critical in the recognition of DSD, but they are slowed down by the system, time, attitudinal, and educational barriers. Providers lack knowledge about the baseline cognitive status of their older adult patients and do not always have access to their family or care partner to assess the acuteness of change [12,25]. Insufficient education and training for clinicians, increased workload, time, and their beliefs about detections and management of DSD versus delirium alone leads to inconsistent application of clinically effective assessments [26,27]. In addition, ageism and potential stigma related to older adults with dementia and confusion about who is responsible for delirium screening are all barriers to assessing and managing delirium [28,29].

Third, the condition DSD and its presentation itself is complicated by a lack of a unifying definition of delirium, overlapping features with dementia, and its variety in presentation [14,30,31]. Finally, patient related factors further add to this challenge as patients with advanced stages of dementia have severe cognitive and functional impairment and, therefore, may already have symptoms of delirium as part of their underlying condition. For instance, the majority of patients with advance stages of dementia experience late-afternoon or early evening increases in confusion, disorientation, and agitation further making it difficult for clinicians to recognize the acute nature of symptoms [32]. In addition, many delirium assessment tools rely on self-report, which may not be possible for someone with advanced dementia who is unable to communicate or is unable to accurately describe their symptoms [32].

In light of the difficulties faced by health care professionals in readily identifying delirium in persons with dementia, policies need to be in place to support and facilitate HCPs in the identification and management of DSD. Research has shown that hospital physical environments can have a significant impact on patient outcomes [33,34]. Noise, limited space to move around and perform basic activities of daily living, and disturbance in sleep can impact the patient’s function and comfort and confound the DSD assessment [26]. Improving these physical setting challenges could potentially improve the clinician’s efforts to conduct accurate assessments, including DSD screening. HCPs who are trained in the use of DSD screening tools and who have access to a supportive practice environment are more likely to be able to accurately detect and manage DSD in their patients. Ensuring that HCPs are “ready” to use DSD screening tools and ongoing appraisal of the practice environment can help to promote the adoption and sustainability of DSD screening practices [35,36]. A more purposeful approach is to regard the brain as a critical organ, and the assessment of brain function should be a part of vital sign assessment in routine clinical care [37,38]. Knowing the older adult is critical, which is crucial for HCPs to provide care that is tailored to the needs and preferences of the individual. Implementing systems or tools, such as electronic health records that allow HCPs to more easily gather, access, and track baseline information about their patient’s mental status, function of their brain, care preferences, and who the important people are in their life that are providing consistent care outside of formal care settings, could potentially improve the assessment of DSD [39]. It is helpful to have processes in place to gather information from family care partners in clarifying the range and context of symptoms of DSD as well as the needs and preferences of their care recipients. In a study by Fick and Foreman, family care partners identified changes in baseline cognitive status in all delirium cases, while only 22% of medical professionals did so [18]. Information obtained from a family care partner, who have often spent more time with the older adult and have known them before hospitalization, can contribute to a more accurate diagnosis of DSD [40,41]. Quality care for an older adult with DSD could be better facilitated by a multidisciplinary team (MDT) approach, where the responsibility of assessment and management of DSD is shared among the hospitalists, nurses, CNAs, and family care partners, which could reduce the workload and burden of assessments on a particular care provider. Studies have found support for the role of CNAs in the assessment and prevention of DSD with appropriate education and training [42,43]. The feeling of shared responsibility and effective communication between the MDT about the status of brain function of the older patient is important to prepare an effective person-centered care plan for the assessment and management of DSD.

## 3. Clinician Tools to Detect DSD

There are several tools that have been developed and validated for the diagnosis of delirium, but performance of these tools in persons with dementia has not been extensively studied, and further research is needed to determine their accuracy in this population. In a systematic review of tools for DSD, Morandi and colleagues identified only nine studies that met their inclusion criteria [23]. In this review, the CAM and CAM-ICU are among the tools that have been more frequently recommended as a reliable and valid tool for the diagnosis of DSD. Persons with dementia were included in both the CAM studies but did not specifically target a dementia subgroup. In a systematic review of 22 studies that included medical and surgical inpatients, the CAM had a pooled sensitivity of 82% (95% confidence interval [CI]: 69–91%) and a pooled specificity of 99% (95% CI: 87–100%) [44]. Only one validation study of the CAM included a high percentage of patients with dementia (85%), but performance of the tool in the different stages of dementia was not discussed [45]. Although CAM may be useful in patients with more advanced stages of dementia who may not be able to communicate verbally, the CAM ratings based on clinician’s observation of patient’s behavior and cognitive function from routine care, and performance relative to a reference standard has been more variable, with some studies reporting a sensitivity as low as 30% [19,46]. Since the Morandi review over a decade ago, several tools have been developed and evaluated in persons with AD/ADRD, but reviewing all in the context of DSD is beyond the scope of this review [23]. Selected tools have been discussed below and are further detailed in Table 1.

Time required to assess for delirium was identified as one of the major barriers in screening for delirium and DSD in patients, and brief testing may be more tolerable [26]. The 3D-CAM, derived from the CAM algorithm can be administered in just 3 min and has a sensitivity of 96% and specificity of 86% when tested in persons with dementia [27]. The Ultra-Brief CAM (UB-CAM) further addresses the time barrier with completion in approximately one minute, is a simple assessment for delirium that uses a combination of two instruments (Ultra-Brief 2 (UB-2)+3D-CAM), is well-accepted by health care professionals, and has been well-validated in persons with dementia [26,52]. In a recent study with 527 older adults, the UB-CAM was feasible (completed by clinicians in over 97% of eligible days), brief (taking on average 1 min 15 s), had an overall accuracy of 89%, and performed equally well when administered by all disciplines, with the lowest accuracy-adjusted cost achieved by RNs [52,53]. The UB-CAM now is available in the form of an apple based iPhone application, which is free and can be downloaded here (https://apps.apple.com/us/app/ub-cam-delirium-screen/id1591656740, accessed on 5 December 2022).

The 4-DSD, modified from the 4-AT, is specifically designed to identify delirium in patients with advanced stage dementia and has a relatively brief administration time (3 min) [49]. The 4-DSD is the only tool that considered the effect of severity of dementia on its performance, showing a decline in specificity with the increasing severity of dementia [49]. However, many of the items in the 4-DSD tool may be attributable to underlying dementia or other cognitive impairment and not to delirium (for instance, unawareness and disturbance in attention and sleep-wake cycle). In cases where a patient has both delirium and dementia, it can be challenging to differentiate the symptoms that are due to delirium from those that are due to the underlying dementia. In such cases, it may be necessary to score the 4-DSD, even if the symptoms can be best attributed to the ongoing dementia process. In addition, it is worth noting that the 4-DSD does not assess all of the symptoms that may be present in both delirium or dementia. For example, agitation, aggression, and psychomotor retardation are not included in the scoring [32]. If these symptoms are present, they may be indicative of delirium or other underlying conditions and may warrant further evaluation.

The 4-AT measures alertness, orientation, and attention and also includes collateral information from family care partners [50]. In the case of the 4-AT, a meta-analysis of 17 studies found that the tool had a pooled sensitivity and specificity of 88% for delirium [54]. However, research has shown that the presence of dementia can adversely affect the accuracy of the 4-AT [50]. In addition, in a study comparing 4-AT with the delirium tool NuDESC (a nurse-based tool), the users felt the 4-AT was harder to fit into a nursing routine and took longer to administer [55]. Research has shown that the 4-AT and the 6-CIT are both effective at excluding delirium and dementia in the emergency department. Both 4-AT and 6-CIT tools require patients to be able to communicate and participate in the cognitive testing, and they have been found to have high sensitivity and specificity in identifying these conditions. However, it is not clear whether a combination of these two tests would increase the specificity for diagnosis of DSD [51].

## 4. Family Care Partner Tools to Detect DSD

As described above, there are a number of tools available for DSD screening now, many of which are based on psychiatric evaluation or numerous subjective observations about acute change by HCPs. The accuracy of these tools is highly dependent on the assessor’s accurate awareness of patients baseline mental status and recent changes. Family care partners are well-suited to provide this crucial information as they know their care recipients well and may have noticed symptoms or behaviors that the patient themselves may not have mentioned or may not have been aware of [12]. Delirium screening instruments exist for capturing the perspective of family care partners or relatives of older patients. These tools (Table 2) have been designated as caregiver-centered tools (tools that can be used by family care partners or nursing assistants without the need for medical training) and caregiver informed tools (tools used by HCPs to get collateral information from family care partners). It is important to carefully consider the properties and limitations of these tools in order to maximize their effectiveness, yet there has been only one review that has compiled and presented the validity of caregiver-centered delirium detection tools frequently used in the hospital settings [41].

Caregiver-informed tools, including the SQiD and SSQ-Delirium, consist of one item, which asks about the presence of confusion or disorientation in the past 24 h. These tools may be highly feasible for use in hospital settings as they are brief and require no training for use by HCPs. These tools could be easily incorporated into HCPs routine care and may potentially improve delirium detection through consistent assessment and documentation of the patient’s mental status and identification of the need for further psychiatric evaluation or referral. However, further validation is required. Family-CAM (FAM-CAM) is a tool designed to provide a method for family care partners to assess for delirium, which is based on the Confusion Assessment Method (CAM) and is a widely used tool for identifying delirium in hospital settings. The FAM-CAM has been validated against the CAM, and previous research has found it to have good sensitivity and specificity of 86% and 98%, respectively [56]. However, a recent study found that the FAM-CAM had moderate sensitivity (57%) and specificity (83%) in identifying delirium [57]. In this study, the FAM-CAM demonstrated a sensitivity of 61% and a specificity of 74% among the dementia subgroup, and the majority of cases where the tool did not correctly identify delirium were due to family care partners not identifying disturbances in attention when using the tool [57]. Despite this caveat, FAM-CAM allows family care partners to report their observations of changes associated with delirium in great detail, for instance, if the changes were acute or if the changes are getting better or worse with time, which is a key feature in distinguishing delirium from dementia in persons with DSD. In contrast to FAM-CAM, the I-AGeD and the Sour Seven tools only include a series of yes/no questions, which may limit the amount of information that can be shared between family care partners and HCPs, often making it difficult to distinguish the symptoms of delirium from pre-existing dementia processes. Since the FAM-CAM has been found to have moderate sensitivity and specificity in some studies, it requires confirmation with a standard delirium rating scale, such as the CAM. However, using structured care partner observations to assess changes in an older adult’s mental status over time and to aid the results of other delirium assessment tools, may help to improve the accuracy of the diagnosis of DSD [32]. One advantage of the FAM-CAM compared to other caregiver-informed tools, such as the Single Question in Delirium (SQiD), which asks “Is this patient more confused than before?”, is that it includes information on additional features of delirium necessary to differentiate delirium from dementia without adding much to the workload of clinicians [58]. This may be helpful in cases when patients are hospitalized due to confusion as their main complaint and in patients with DSD who have confusion as part of their underlying dementia [57].

**Table 2:** Family care partner delirium/delirium superimposed on dementia detection tools.

Delirium Screening Tools	Caregiver Administered or Informed	Age (Mean/SD)	Total Sample	Setting	Sample with Delirium (N, %)	Sample without Delirium or Dementia (N, %)	Sample with Dementia (N, %)	Sample with Delirium Superimposed on Dementia(N, %)	Reference Standard	Sensitivity % (95% CI)	Specificity % (95% CI)
Single Question in Delirium (SQiD) [58]	Informed	Primary analysis 68 (60.5–78); Secondary analysis 68 (56–77.5).	Background population = 2353; Analyzed group for DSM = 73; Analyzed group for CAM = 122.	Inpatient oncology wards	NA	NA	NA	NA	DSM-IV and CAM	44.4(25.5–64.7%)	87(74–95%)
Single Question-Delirium (SSQ-Delirium) [59]	Informed	79.6 (65–97)	161	Acute medicine unit/geriatric assessment unit	9(12.9%)	25(35.7%)	10(14.3%)	26(14.1%)	CAM	76.9%	56.1%
Family CAM [57]	Administered	Patient 80.3 ± 7 FC 80.3 ± 6.9	108 family and caregiver dyad	Emergency department	30(28%)	45(42%)	55(51%)	22(20%)	CAM	Overall 56.7(37–75%); Dementia subgroup 60.8(41–81%); No Dementia subgroup 42.8(6–80%).	Overall 83.3(75–92%); Dementia subgroup 74.3(60–89%); No Dementia subgroup 90.7(82–99%).
Informant Assessment of Geriatric Delirium [60]	Administered	85.5 ± 7.6	85	Nursing home	5(5.9%)	47(58.8%)	35(41.2%)	2(40%)	DSM-5	82 (60%); Cut off score 4.	64 (94%); Cut off score 4.
Sour Seven [61]	Administered	81.3 ± 8.9	39 (analyzed group)	Medical and surgical units	NA	NA	NA	NA	DSM-5	Total score of 4 89.5%; Total score of 9 63.2%.	Total score of 4 90%; Total score of 9 100%.

In summary, using these tools, family care partners may be able to more readily identify symptoms of delirium or DSD than HCPs who are often not aware of their patient’s baseline mental status. In addition, using caregiver-centered delirium detection tools may provide a way for continuous screening for DSD, which might otherwise be missed during intermittent screening because of its fluctuating course. However, these caregiver- centered tools should not be used as a stand-alone test to screen for DSD. In older adult patients with dementia who are at a high risk for developing delirium during a hospital stay, it would be feasible for HCPs to ask their family care partner the single question about change in the patient’s mental status or provide a copy of the FAM-CAM tool to complete to get more detailed information about changes in the mental status of patients during their hospital stay. The results of the tool provided could then be used to inform further clinician assessments for DSD or referral for a full psychiatric evaluation as necessary. An additional benefit of the caregiver administered tools is that family care partners can screen for delirium or DSD throughout the patient’s hospital stay without any assistance and notify HCPs when changes occur. It may empower FCPs to assess for delirium or DSD in their care recipients at home settings and seek appropriate care when changes occur. In addition, engaging FCPs in delirium or DSD detection may potentially alleviate their distress and anxiety associated with witnessing sudden changes in the mental status of their care recipient and may improve caregiver satisfaction with patient care [56]. Hospital staff should also educate family care partners of at-risk older adult patients about DSD and direct them to the resources in Table 3 for additional information.

## 5. Discussion and Implications

Delirium in persons with dementia is a common occurrence. Over the past three decades, several tools have been developed and validated to diagnose delirium, yet there is still a shortage of tools recommended in persons with dementia and a lack of sufficient research on the accuracy of performance of such tools in this growing population (see Table 1). The most important finding of this review is that despite having many delirium tools, only a few have been thoroughly tested in persons with dementia and though there are barriers to detection, many of these are modifiable. The NIDUS website has data on 25 delirium tools and the review by Han and colleagues reviewed more than 40 delirium instruments, recommending the CAM as the top delirium instrument, yet other studies have shown that CAM does not translate as well at the bedside [7,19,46]. A review by Brefka and colleagues also point out the difficulties of using the CAM in clinical settings or with untrained individuals and in persons with dementia [62]. The 4-AT is one of the most widely used tools, but the presence of acute change is not necessary for a positive delirium and has not been well tested in persons with dementia [63]. Most importantly, the specificity of the 4-AT tool has been found to be considerably decreased in persons with dementia [50,51]. Our review (Table 1) examined seven tools in depth and only a few of them included adequate numbers of persons with dementia in their studies. This review did not answer the question of “which tool is best in DSD?” as the tools had both strengths and limitations, and all have room for improvement. In a clinical setting, the best tool is the one that fits the needs of your clinical setting, resources, and context. The instruments that had better performance in persons with DSD when including a larger sample of persons with dementia include 4-DSD and UB-CAM. When taken into consideration the severity of dementia, 4-DSD fared better with high sensitivity and specificity in persons with moderate to severe dementia, whereas other tools have not been tested well enough in different dementia subgroups [49]. The UB-CAM, however, was quicker at providing an assessment for delirium in approximately one minute, has been shown to have good reliability and validity in identifying delirium in hospitalized patients, including those with dementia, and is well-accepted by the HCPs [52]. In terms of family caregiver tools for the detection of DSD, based on our review, FAM-CAM is the only tool recently tested specifically in a dementia group and performed fairly well in terms of sensitivity and specificity in persons with dementia [57]. Compared to delirium detection tools for use by HCPs in persons with dementia, considerable progress needs to be made in the development, validation, and replication of family/caregiver-assisted/informed DSD detection tools. Using a combination of delirium detection tools used by HCPs and FCPs could potentially improve the detection of DSD. Fong and colleagues recently proposed an approach that uses a modified CAM to perform a more structured assessment of the acute change in mental status with input from a family care partner or nurse. This approach importantly raises issues of the difficulty of assessing acute change in advanced dementia, takes 15–20 min, relies on having an informant, and has not yet been tested [32]. This needs to go beyond the acute care setting as work by Shrestha shows and this was highlighted during the pandemic with the increase in delirium, both in the home and hospital setting [12]. Along with the tools to detect delirium in persons with dementia, some small studies have proposed assessing brain electrical activity as a useful method in the diagnosis of delirium. A study by Thomas and colleagues performed electroencephalogram (EEG) tests on a small sample of 35 older adults with dementia admitted to an acute geriatric ward and found the test to have moderate sensitivity (67%; quantitative EEG (eyes open)) in identifying DSD [64]. The utility of an EEG in detecting DSD needs further study in a larger sample of older adults with dementia along with a consideration for the different types and severity of pre-existing dementia to improve widespread clinical applicability.

We have made strong progress over the past three decades, yet much work remains to be done in the area of DSD to improve detection, and even more urgently to improve the implementation of detection in a busy clinical setting for persons with dementia that are at the highest risk for delirium. Importantly many of the barriers found in this review are potentially modifiable in practice and research with the right system changes, policies, better inclusion of family and care partners (i.e. to address the issue of baseline) and careful attention to instrument design and implementation that addresses time, workload fit (especially for bedside nurses who most often screen for delirium), integration of tools into the electronic record and dashboard, and increased education. In this review, even those tools that included persons with dementia had very few participants with advanced dementia. As Fick points out in her recent commentary, we lack understanding of many aspects of DSD that requires more work and innovation, “how many with severe dementia develop delirium, and how delirium may present differently in advanced dementia or different types of dementia. We also need to explore more what constitutes recovery in DSD, the continuum of delirium and dementia and overlapping symptoms, and whether other measures such as biomarkers, eye movement, posture or facial expression, or other manifestation may be useful” p. 1080 [39]. Clearly, more work is needed in better understanding DSD and optimizing and standardizing feature assessment, especially the acute change feature at the bedside for DSD. Larger pragmatic trials under real-world conditions are needed and future artificial intelligence tools with virtual observation of persons with delirium may help advance the science in this area.

Given this review, an “ideal” tool for DSD would point to tools being brief, easy to integrate into the EMR, and accurate with at least 90% accuracy given the poor outcomes associated with delirium and DSD. In addition, knowing the baseline and communication between family care partners and HCPs should be a top priority for education, research, and health systems policy. The detection and management of DSD should be a shared responsibility and should be tackled with a multidisciplinary approach rather than wondering about whose responsibility it is. Systematic changes in health system policy should be made to create a culture and environment that prioritizes assessment of mentation as part of routine clinical care and involves family care partners in improving patient outcomes. In addition, ongoing education and training for HCPs and family care partners about DSD, its detection, management, and available tools should be provided. Since delirium occurs most commonly in persons with dementia, research should prioritize studies of delirium tools and measures specific to DSD. Table 4 outlines an agenda for research, education, and policy to improve the detection of DSD that addresses these issues as important for future work. This study has few limitations. This study is a clinical review and not an exhaustive systematic review. Hence, all screening tools used by HCPs or family care partners for detection of DSD have not been reviewed. In addition, various underlying dementia causes, the underlying neurodegenerative pathology, and the possibility of complicating psychiatric diseases, which could confound delirium assessment in persons with DSD, have not been included. Future work should consider the underlying causes and types of dementia along with complicating psychiatric disorders, such as depression, when developing and testing tools for DSD.

## Figures and Tables

**Table 1:** Health care professional delirium/delirium superimposed on dementia detection tools.

Tools (Author, Year)	Age (Mean ± SD)	Setting	Total Sample (N)	Sample with Delirium N(%)	Sample without Delirium or Dementia N(%)	Sample with Dementia N(%)	Sample with Delirium Superimposed on Dementia N(%)	Sample with Depression N(%)	Sensitivity of the Tool in the Entire Sample	Specificity of the Tool in the Entire Sample
CAM [47]	77–81 ± 5.4–7.9	Internal medicine service	56	26(46%)	27(48%)	12(21%)	9(16%)	9 (16%)	94–100%	90–95%
CAM-ICU [48]	55.3 ± 17.4	ICU	96	80(83%)	15(16%)	12(15%)	11(11%)	30(29%)	93–100%	98–100%
3D-CAM [27]	84 ± 5.5	General medicine units	201	42(21%)	NA	56(27%)	28(14%)	NA	Entire sample 95(84–99%); Dementia subgroup 96(82–100%); No Dementia subgroup 93(66–100%).	Entire sample 94(90–97%); Dementia subgroup 86(67–96%); No Dementia subgroup 96(91–99%).
UB-CAM [42]	79.7 ± 6.6	General medicine inpatients	527	114(27%)	303(57%)	183(35%)	73(64%)	NA	Physician 56.3(35.4–76.1%); Nurse 64.4 (41.6–82.3%).	Physician 93.1(85.0–96.8%); Nurse 93.9 (86.7–97.2%).
4-DSD [49]	85.32 ± 5.79	Acute hospital and rehabilitation setting	134	NA	NA	134(100) Or 46(32%)	Acute hospital 37(80.43); Rehabilitation setting 9(19.57).	NA	Entire sample 80(66–91%); Moderate to severe dementia 79(60–92%); Severe dementia 82(57–96%).	Entire sample 80(70–87%); Moderate to severe dementia 82(72–90%); Severe dementia 56(21–86%).
4-AT [50]	83.9 ± 6.1	Acute geriatrics ward and a department of rehabilitation.	234	29 (12%)	150(63%)	74(31%)	17(7%)	NA	Entire sample (89.7%); Dementia subgroup (94.1%); No Dementia subgroup (83.3%).	Entire sample (84.1%); Dementia subgroup (64.9%); No Dementia subgroup (91.3%).
6-CIT +4-AT [51]	77 ± *N*/*A*	Emergency department	419	15.2%	NA	21.5%	49.4%	NA	89.9%	62.7%

**Table 3:** Selected Resources and Education for Family Care Partners.

Resource	URL
Videos Addressing Brain Health Issues in Hospitals, Emergency Rooms (AARP)	https://www.aarp.org/health/brain-health/global-council-on-brain-health/hospital-emergency-care/ (accessed on November 2022)
Mentation: Recognizing Dementia, Delirium, and Depression (AARP)	https://videos.aarp.org/detail/video/6263557225001/mentation:-recognizing-dementia-delirium-and-depression (accessed on November 2022)
GCBH Recommendations to Prevent and Treat Delirium (AARP)	https://www.aarp.org/content/dam/aarp/health/brain_health/2020/03/gcbh-delirium-report-english.doi.10.26419-2Fpia.00101.001.pdf (accessed on November 2022)
Family Guidance (American Delirium Society)	https://americandeliriumsociety.org/patients-families/family-guidance/ (accessed on November 2022)
Age-Friendly Health System (IHI)	https://www.ihi.org/Engage/Initiatives/Age-Friendly-Health-Systems/Pages/default.aspx (accessed on November 2022)
Patient & Family Resources (HELP)	https://help.agscocare.org/table-of-contents/Patient-Family-Resources/H00109 (accessed on November 2022)
Family CAM toolkit (AGS CoCare)	https://help.agscocare.org/chapter-abstract/chapter/H00101/H00101_PART001_005 (accessed on November 2022)
Family caregiving for delirious patients: what it’s like, what we know, and what’s next (NIDUS)	https://deliriumnetwork.org/family-caregiving-for-delirious-patients/ (accessed on November 2022)
Understanding Delirium (ADS)	https://www.youtube.com/watch?v=M4wsPTtGeIc (accessed on November 2022)

**Table 4:** Research, Policy, and Education Agenda for Detection of Delirium Superimposed on Dementia in Older Persons.

Engaging family caregivers in assessment of DSD
Develop educational programs including materials and brochures to educate family caregivers about the causes, risk factors, pathologies, overlapping features of delirium and dementia, expected course, universality of DSD, preventable nature of delirium, and nonpharmacological strategies in the prevention and management of DSD. Utilize teach-back method with family caregivers to know what they have understood to address misunderstandings and concerns. Utilize family caregivers at the bedside or at home to report deviation in cognition and behavior in the patient using simple screens like FAM-CAM with appropriate training. Assess the cost-effectiveness of inclusion of family caregivers in the recognition and management of DSD. Explore the potential advantage of utilizing nonpharmacological interventions early on the prevention of DSD at home. Explore the potential use of technology to improve communication with health care professionals and timely reporting of acuteness of change in cognition. Explore the potential barriers and facilitators to detection and reporting of acute change in PLWD by family caregivers.
Engaging health care professionals in assessment of DSD
Develop educational programs and provide periodic in-service education about DSD, features of delirium, DSD, dementia, and overlaps, risk factors, assessment tools, importance of early detection, prevention, and management of DSD. Emphasize the importance of routine screening for delirium/DSD among all hospitalized older adults patients. Explore potential of inclusion of certified nursing assistants in routine screening of older adults with dementia for delirium using tools like UB-2 with appropriate training followed further evaluation by nurse or clinician. Provide short and sensitive delirium detection tools that are integrated in the EMR for continuous tracking of appropriate care and patient outcomes. Continue to elicit the voice of health care professionals in the challenges of assessment of DSD across countries. Incorporate delirium/DSD into annual competency assessment.
Health system & policy issues
Establish delirium/DSD detection, prevention, and management as a shared responsibility among hospital staff by implementing a multidisciplinary approach. Establish an evidence-based streamlined organization protocol for prevention, screening, reporting, and management of DSD. Integrate of delirium/DSD into 4-M Age Friendly Care dashboard and IT systems build like Cerner and EPIC Consider global pathways for delirium and dementia. Integrate mentation screening into hospital protocols and standardized order sets to establish mentation screening as a part of routine screening across settings of care. Address the stigma and ageism around caring for persons with dementia and delirium. Provide Information technology support, payment incentives, and supportive work culture around the detection of delirium/DSD. Establish a national or global pathway for delirium and dementia.
Research design
Develop and refine diagnostic criteria and instruments for delirium superimposed on dementia. Methodological issues to consider: — Identify overlapping features of delirium superimposed on dementia with defined measures; — Distinguish important clinical features of delirium with dementia versus exacerbation of existing dementia and behavioral disturbances; — Elucidate factors that determine recognition of delirium in persons with dementia. — Consider different phenotypes of delirium; — Consider neuropathological and in-depth studies to better understand DSD as a unique phenotype; — Develop valid methods and measures to define premorbid functioning if history is not known or family/caregivers not available. Replication of validation studies in large sample sizes including persons with dementia and their family caregivers. Design studies that oversample persons with moderate to severe dementia. Consider incorporating bio-physical measures. Convene patient and family advisory boards that help in design, conduct, and dissemination of studies of delirium/DSD detection. Have implementation in mind when designing studies agile implementation, step wedge design, SMART design. Design studies that link detection to outcomes important to older adults, their caregivers and health system. Establish core set of measures for how we screen for dementia in studies for delirium. Standardize how we assess the acute features of delirium in persons with dementia with the help of family care partners and formal and informal caregivers. Assure diversity of the sample and consider using young older adults with dementia. Establish practical guidelines for consent and studies of older persons with cognitive impairment. Issues to consider: — Identify the best measures for obtaining proxy consent and assent; — Develop methods to ensure the right of the person with dementia to refuse participation at any time; — Consider when and how to notify clinicians of study results.

## Data Availability

No new data were created or analyzed in this study. Data sharing is not applicable to this article.
